# Prevalence and factors associated with poor self-assessed health in Brazilian adolescents: National School Health Survey 2019

**DOI:** 10.1590/1980-549720260007

**Published:** 2026-03-16

**Authors:** Luís Antônio Batista Tonaco, Bárbara Aguiar Carrato, Leonardo Lemos Pena, Deborah Carvalho Malta

**Affiliations:** I Universidade Federal de Minas Gerais - Belo Horizonte (MG), Brazil.; II Universidade Federal de Minas Gerais, School of Nursing - Belo Horizonte (MG), Brazil.; III Universidade Federal de Minas Gerais, School of Nursing, Graduate Program - Belo Horizonte (MG), Brazil.; IV Universidade Federal de Minas Gerais, School of Nursing, Department of Maternal-Child and Public Health Nursing - Belo Horizonte (MG), Brazil.

**Keywords:** Adolescent, Self-testing, Health status, Health surveys, Brazil

## Abstract

**Objective:**

To estimate the prevalence and factors associated with poor self-assessed health among Brazilian school-aged adolescents aged 13 to 17 years.

**Methods:**

Cross-sectional study using data from the 2019 *National School Health Survey* (PeNSE). Crude and adjusted prevalence ratios (APR) were estimated by logistic regression. Analyses were performed in *Stata* 16.

**Results:**

Factors associated with poor self-rated health were: being male (APR 0.75; 95%CI 0.72-0.79); aged 16-17 years (APR 1.13; 95%CI 1.08-1.18); Black (APR 1.08; 95%CI 1.01-1.14), Brown (APR 1.10; 95%CI 1.05-1.16), or other (APR 1.13; 95%CI 1.04-1.22); eating breakfast (APR 0.84; 95%CI 0.80-0.87); consumed alcoholic (APR 1.12; 95%CI 1.07-1.18); perceiving oneself as overweight (APR 1.34; 95%CI 1.27-1.42) or underweight (APR 1.12; 95%CI 1.06-1.18); engaging in >300 minutes of physical activity per week (APR 0.85; 95%CI 0.80-0.89); consuming fruits (APR 0.86; 95%CI 0.81-0.92); having one or more friends (APR 0.80; 95%CI 0.74-0.86); having ever sought health services (APR 0.92; 95%CI 0.88-0.96); missing school due to health problems for 1-3 days (APR 1.31; 95%CI 1.24-1.39) or ≥4 days (APR 1.64; 95%CI 1.54-1.74); feeling worried (APR 1.09; 95%CI 1.02-1.15), sad (APR 1.33; 95%CI 1.24-1.41), that no one cares about them (APR 1.22; 95%CI 1.16-1.29), irritated (APR 1.19; 95%CI 1.10-1.25), or that life is not worth living (APR 1.38; 95%CI 1.32-1.45).

**Conclusion:**

Sociodemographic, behavioral, and mental health factors were associated with poor self-rated health among Brazilian adolescents.

## INTRODUCTION

Self-assessment of health (SAH) is widely used in population-based studies and surveys as a key subjective indicator of health and well-being, given its ease of application and its capacity to capture individual perceptions of health status^1^ ^,^ ^2^ ^,^ ^3^ ^,^ ^4^. Although subjective in nature, SAH represents a comprehensive measure that reflects both perceived health status and the influence of social, behavioral, and environmental determinants^1^ ^,^ ^2^ ^,^ ^3^ ^,^ ^4^. Recent studies indicate that SAH is associated with multiple objective and subjective factors, including socioeconomic conditions (education, income, occupation), as well as cultural, psychological (such as the presence of depression or subjective well-being), and social aspects (including support networks and interpersonal relationships)^1^ ^,^ ^2^ ^,^ ^3^ ^,^ ^4^.

A positive perception of one’s own health is associated with psychological well-being^5^, greater adherence to treatments, and the adoption of preventive behaviors, resulting in improved health outcomes^6^. Conversely, a negative self-perception is linked to poorer prognoses, increased substance use, and the adoption of harmful behaviors, thereby elevating health risks^7^ ^,^ ^8^. Thus, self-assessment is considered an important tool for analyzing population health conditions and supports the formulation and implementation of public health promotion policies^7^.

In recent decades, self-perceived health among adolescents has gained prominence in research addressing the determinants of the health-disease process^9^ ^,^ ^10^. Adolescence constitutes a period of intense physical, psychological, and social transformation, marked by the development of individual and collective identity, which directly influences the adoption of habits and behaviors that may persist into adulthood^11^ ^,^ ^12^ ^,^ ^13^. Evidence indicates that older female adolescents, those who perceive themselves as overweight, or those who exhibit symptoms of psychological distress tend to report poorer self-assessed health. In contrast, adolescents who maintain healthy habits, such as eating breakfast regularly, engaging in physical activity, and consuming fruit, demonstrate a more favorable perception of their own health^4^ ^,^ ^11^ ^,^ ^12^ ^,^ ^13^ ^,^ ^14^ ^,^ ^15^.

Several factors have been associated with a negative perception of health in this age group, including risky behaviors such as physical inactivity, smoking, alcohol consumption, and the use of other substances, as well as exposure to school violence^11^ ^,^ ^13^ ^,^ ^14^. Sociodemographic characteristics, such as sex, age, cohabitation with parents, participation in family meals, and frequency of breakfast consumption, are also linked to higher prevalences of negative self-perceived health among adolescents^4^ ^,^ ^15^.

In international contexts, similar findings have been reported across different countries, indicating consistent associations between negative self-perceived health and factors such as stress, depressive symptoms, low levels of physical activity, and inadequate eating habits^16^. In South Korea, for example, a study conducted with elementary and high school students demonstrated that negative self-perceived health was significantly associated with stress, suicidal ideation and attempts, low physical activity, and skipping meals such as breakfast^16^. These findings align with observations in other populations, reinforcing that, regardless of sociocultural context, psychosocial and behavioral determinants play a central role in how adolescents perceive their health.

In the Brazilian context, the National School Health Survey (*Pesquisa Nacional de Saúde do Escolar* - PeNSE) has incorporated SAH into its questionnaires, recognizing its relevance for understanding health perception among adolescents^4^. An analysis of PeNSE 2015 data identified that girls, adolescents aged 16 and 17, those self-identified as Black or Brown, and those engaging in risk behaviors had a higher likelihood of poor SAH. In contrast, regular breakfast consumption and engagement in physical activity were associated with a lower likelihood of negative self-perception, although the definition of the outcome variable differed from that adopted in the present study^4^. Other national surveys have likewise demonstrated associations between SAH and a range of sociodemographic and lifestyle characteristics, including quality of life^4^, sex^4^ ^,^ ^10^, age^4^ ^,^ ^10^, race/color^4^, lifestyle habits^4^ ^,^ ^10^, health service utilization^4^, and maternal education^4^ ^,^ ^15^.

Despite notable advances, a gap remains in the scientific literature regarding the assessment of negative self-perceived health among Brazilian adolescents in nationwide studies using up-to-date data. Measuring SAH in population surveys through standardized scales enables a representative appraisal of adolescents’ health perceptions and provides essential support for understanding the social and behavioral determinants that influence well-being^4^.

Therefore, given the relevance of SAH as an indicator of adolescents’ health conditions and psychological well-being, the present study aimed to estimate the prevalence of, and factors associated with, poor self-assessed health status among Brazilian schoolchildren aged 13 to 17 years, using data from the 2019 PeNSE.

## METHODS

This is a cross-sectional study based on data from the 2019 edition of PeNSE. The data are publicly available on the website of the Brazilian Institute of Geography and Statistics (*Instituto Brasileiro de Geografia e Estatística* - IBGE)^17^.

PeNSE is a national survey conducted by IBGE, in partnership with the Ministry of Health and with support from the Ministry of Education^17^ ^,^ ^18^. It investigates risk and protective factors for the health of Brazilian adolescents, collecting information on behaviors such as physical activity, diet, substance use, oral health, sexual behavior, and exposure to violence^17^ ^,^ ^18^. The first edition of PeNSE was carried out in 2009 and was subsequently repeated in 2012, 2015, and 2019^17^ ^,^ ^18^.

The final sample consisted of students enrolled in the selected classes. The survey was based on the Registry of Public and Private Schools of the National Institute of Educational Studies and Research Anísio Teixeira (*Instituto Nacional de Estudos e Pesquisas Educacionais Anísio Teixeira* - Inep), ensuring representativeness at the following geographic levels: Brazil, major regions, states, state capitals, and the Federal District^17^ ^,^ ^18^.

All students regularly enrolled in, and attending, classes from the 7^th^ to the 9^th^ grade of elementary school and from the 1^st^ to the 3^rd^ year of high school in public and private institutions-across morning, afternoon, and evening shifts-were selected to complete the questionnaire^17^ ^,^ ^18^. Students enrolled in technical programs integrated with high school and in teacher training courses were also included^17^ ^,^ ^18^. Only students present on the day of data collection participated in the survey. Schools with fewer than 20 enrolled students were excluded^17^ ^,^ ^18^.

The sample was obtained through multi-stage (cluster) probability sampling, stratified by state, location (urban or rural), and type of school (public or private) ^17^ ^,^ ^18^.

The selection process was conducted in three stages:

1. Probabilistic selection of schools within each stratum;
2. Random selection of classes in the selected schools; and
3. Inclusion of all students who were present in the selected classes at the time of data collection^17^ ^,^ ^18^.

Further methodological details regarding the study and the sampling plan are available in previous publications^17^ ^,^ ^18^.

For this analysis, the outcome of interest was SAH status, based on the question included in the 2019 PeNSE questionnaire: “*How would you rate your health status?*”. Response options were: very good, good, fair, poor, and very poor. To construct the outcome variable (poor SAH), the responses were dichotomized into two categories: the first combined “very good” and “good” whereas the second grouped “fair,” “poor,” and “very poor.”

SAH is commonly measured using a single item with a five-point ordinal scale, similar to that employed in PeNSE. Terms such as *self-rated health*, *self-assessed health*, and *self-reported health* are frequently used interchangeably in the literature to denote this subjective measure of perceived overall health^4^ ^,^ ^17^ ^,^ ^18^. The question used to assess SAH is widely applied in population-based surveys and has well-established evidence of validity and reliability across different contexts^4^ ^,^ ^17^ ^,^ ^18^.

The independent variables were selected based on the literature, which indicates that self-perceived health is associated with sociodemographic factors, level of education, race/color, family characteristics, lifestyle habits, health-related behaviors, use of health services, and mental health indicators^4^. Accordingly, the independent variables were distributed as described below.

Sociodemographic characteristics included gender (female; male), age in years (13-15; 16-17 years), race/color (white; Black; Brown; other), type of school (public; private), and maternal education (no schooling; primary education [incomplete/complete]; secondary education [incomplete/complete]; and higher education [incomplete/complete]). Family characteristics included living with the mother (yes; no), living with the father (yes; no), and the frequency of eating meals with a caregiver (none; ≤2 times per week; 3-4 times per week; ≥5 times per week).

Regarding lifestyle and behavioral characteristics, the following variables were considered: regular breakfast consumption (did not eat; ≥5 times per week; 1-4 times per week), ever having tried cigarettes (no; yes), ever having tried alcoholic beverages (no; yes), ever having tried drugs (no; yes), consumption of soft drinks >5 times per week (no; yes), consumption of fruit >5 times per week (no; yes), engagement in regular physical activity (<300 minutes per week; ≥300 minutes per week), and history of sexual intercourse (no; yes).

Furthermore, the analysis considered whether the adolescent had ever missed school for health-related reasons (none; 1-3 days; ≥4 days), their self-perceived body image (normal; overweight; underweight), and whether they had ever sought health services (no; yes). Mental health characteristics included having friends (none; one or more); feeling worried in the past 30 days (no; yes); feeling sad in the past 30 days (no; yes); feeling that nobody cares about them in the past 30 days (no; yes); feeling irritated, nervous, or moody in the past 30 days (no; yes); and feeling that life is not worth living in the past 30 days (no; yes).

Initially, descriptive analyses were conducted to estimate prevalences and their respective 95% confidence intervals (95% CI) according to the independent variables described above.

Subsequently, to investigate factors associated with the outcome of interest (poor SAH), a bivariate analysis was first conducted to estimate unadjusted prevalence ratios (PR) and their respective 95%CIs using simple logistic regression. Variables with p < 0.20 in the bivariate analysis were then included in a multivariate logistic regression model. In the final adjusted model (PRa), only variables with statistical significance (p < 0.05) were retained. Model fit was assessed using the Hosmer-Lemeshow test.

All analyses were performed using the svy module of Stata Statistical Software, version 16, which allows for the appropriate incorporation of sample weights, strata, and clusters.

This study utilized secondary data, thereby exempting it from submission to a Research Ethics Committee. However, the 2019 edition of PeNSE received approval from the National Research Ethics Committee on Human Beings (*Comissão Nacional de Ética em Pesquisa com Seres Humanos* - Conep) under opinion No. 3.249.268^17^ ^,^ ^18^.

## DATA AVAILABILITY DECLARATION

The entire dataset supporting the results of this study is available upon request from the corresponding author.

## RESULTS

Of the total 103,177 adolescents included in the database, 100,221 (97.1%) had complete information on the variables of interest and were included in the final analysis. A total of 2,956 cases (2.9%) were excluded due to missing data for one or more variables.

The study sample was composed predominantly of female students (52.17%), aged 13-15 years (64%), and of mixed race (43.01%). The majority were enrolled in public schools (51.73%) and had mothers with incomplete or complete higher education (46.91%). Regarding lifestyle habits, 58.95% of students reported regularly eating breakfast, 79.63% had never tried cigarettes, 63.94% had tried alcoholic beverages, and 87.59% had never tried drugs. Frequent consumption of soft drinks was reported by 15.92% of students, and frequent fruit consumption by 28.42%. In terms of physical activity, 71.58% engaged in less than 300 minutes per week (Table 1).

**Table 1.** Distribution and prevalence of poor self-assessment of health among Brazilian school adolescents, according to associated factors, with corresponding 95% Confidence Intervals. National School Health Survey, Brazil, 2019.

	Distribution (%)	Fair/poor/very poor	p-value
		n (%)	
Total		32.80 (32.51-33.08)	
Gender			
Female	52.17	39.55 (38.62-40.50)	<0.0001
Male	47.82	20.96 (20.17-21.77)	
Age (years)			
13 to 15	64.00	28.15 (27.27-29.04)	<0.0001
16 or 17	35.99	35.69 (34.75-36.63)	
Race/skin color			
White	40.32	28.93 (27.81-30.07)	0.0008
Black	10.47	30.43 (28.79-32.13)	
Brown	43.01	31.86 (30.75-33)	
Others	6.18	32.99 (30.42-35.68)	
Type of school			
Public	51.73	31.10 (30.33-31.88)	<0.0001
Private	48.26	28.11 (27.33-28.91)	
Mother’s education level			
None/Primary (incomplete/complete)	22.5	30.88 (29.80-31.98)	0.0019
Secondary (incomplete/complete)	30.57	31.97 (30.80-33.17)	
Higher education (incomplete/complete)	46.91	28.84 (27.62-30.10)	
Regular breakfast consumption			
Does not eat breakfast	32.42	39.60 (38.43-40.78)	<0.0001
1 to 4 times per week	8.79	34.61 (32.23-37.07)	
5 or more times per week	58.95	25.16 (24.43-25.91)	
Ever smoked			
No	79.63	28.07 (27.31-28.84)	<0.0001
Yes	20.36	39.70 (38.23-41.20)	
Ever drank alcohol			
No	36.05	22.75 (21.89-23.64)	<0.0001
Yes	63.94	35.22 (34.30-36.14)	
Drug experimentation			
No	87.59	29.11 (28.53-29.80)	<0.0001
Yes	12.4	41.31 (39.37-43.28)	
Body image			
Normal	47.91	24.35 (23.41-25.32)	<0.0001
Overweight	23.30	44.28 (42.57-46)	
Underweight	28.77	31.50 (30.31-32.72)	
Soft drink consumption (>5 times/week)			
No	84.07	30.27 (29.48-31.07)	0.019
Yes	15.92	32.56 (30.95-34.22)	
Frequent fruit consumption (>5 times/week)			
No	71.57	32.87 (31.98-33.77)	<0.0001
Yes	28.42	25.05 (23.77-26.37)	
Regular physical activity (minutes per week)			
<300	71.58	33.54 (32.69-34.40)	<0.0001
>300	28.41	23.71 (22.70-24.75)	
Friends			
None	3.20	44.69 (41.23-48.21)	<0.0001
1 or more	96.79	30.10 (29.40-30.80)	
Sought any health service			
No	35.95	29.19 (28.13-30.28)	0.0002
Yes	64.04	31.73 (30.91-32.55)	
Missed class due to health problems			
No	37.72	23.40 (22.54-24.29)	<0.0001
1 to 3 days	52.31	33.89 (32.79-35.01)	
4 or more days	9.95	47.84 (45.39-50.30)	
Felt worried in the past 30 days			
No	15.53	23.63 (22.25-25.07)	<0.0001
Yes	84.46	32.53 (31.81-33.26)	
Felt sad in the past 30 days			
No	31.87	16.19 (15.35-17.06)	<0.0001
Yes	68.12	38.12 (37.30-38.94)	
Felt that no one cares about you in the past 30 days			
No	45.88	18.10 (17.05-19.21)	<0.0001
Yes	54.11	35.07 (34.33-35.82)	
Felt irritated, nervous, or in a bad mood in the past 30 days			
No	23.09	18.10 (17.05-19.21)	<0.0001
Yes	76.9	35.07 (34.33-35.82)	
Felt that life is not worth living in the past 30 days			
No	63.89	21.43 (20.70-22.18)	<0.0001
Yes	36.1	45.82 (44.78-46.85)	

95%: 95% confidence interval.

The predominant self-perceived body image was normal (47.91%). Nearly all adolescents reported having one or more friends (96.79%), 84.46% had sought some type of health service, and 52.31% had missed school for four or more days due to health-related reasons. Regarding psychological well-being, 84.46% reported feeling worried; 68.12% felt sad; 54.11% felt that nobody cared about them; 76.90% felt irritated, nervous, or moody; and 36.10% felt that life was not worth living (Table 1).

Among Brazilian schoolchildren, 32.80% (95%CI 32.51-33.08) reported poor SAH. This prevalence was higher among females (39.55%; 95%CI 38.62-40.50), adolescents aged 16-17 years (35.69%; 95%CI 34.75-36.63), those of non-white race/color (32.99%; 95%CI 30.42-35.68), students enrolled in public schools (31.10%; 95%CI 30.33-31.88), and those whose mothers had incomplete/complete secondary education (31.97%; 95%CI 30.87-33.17). Higher prevalences were also observed among schoolchildren who did not regularly eat breakfast (39.60%; 95%CI 38.43-40.78), those who had tried cigarettes (39.70%; 95%CI 38.23-41.20), and those who had consumed alcoholic beverages (35.22%; 95%CI 34.30-36.14) (Table 1).

Higher prevalence rates were also observed among students who had experimented with drugs (41.31%; 95%CI 39.37-43.28), those who perceived themselves as overweight (44.28%; 95%CI 42.57-46.00), those who regularly consumed soft drinks (32.56%; 95%CI 30.95-34.22) and did not consume fruit with the same frequency (32.87%; 95%CI 31.98-33.77), and those engaging in less than 300 minutes of physical activity per week (33.54%; 95%CI 32.69-34.40). Higher prevalences were also reported among adolescents without friends (44.69%; 95%CI 41.23-48.21), those who had sought health services (31.73%; 95%CI 30.91-32.55), and those who had missed school for health reasons for four or more days (47.84%; 95%CI 45.39-50.30) (Table 1).

Furthermore, higher prevalences were observed among schoolchildren who, in the last 30 days, reported feeling worried (32.53%; 95%CI 31.81-33.26); sad (38.12%; 95%CI 37.30-38.94); that nobody cares about them (35.07%; 95%CI 34.33-35.82); irritated, nervous, or moody (35.07%; 95%CI 34.33-35.82); and that life is not worth living (45.82%; 95%CI 44.78-46.85) (Table 1).

Table 1 of the Supplementary Material presents the estimates from the crude model (Table 1S). After adjusting for all variables in the model, the characteristics of schoolchildren that remained associated with poor self-rated health were: being male (PRa 0.75; 95%CI 0.72-0.79); aged 16-17 years (PRa 1.13; 95%CI 1.08-1.18); Black race/color (PRa 1.08; 95%CI 1.01-1.14), Brown (PRa 1.10; 95%CI 1.05-1.16), and other race/color (PRa 1.13; 95%CI 1.04-1.22); eating breakfast five or more times per week (PRa 0.84; 95% CI 0.80-0.87); having previously tried alcoholic beverages (PRa 1.12; 95%CI 1.07-1.18); and perceiving oneself as overweight (PRa 1.34; 95%CI 1.27-1.42) or underweight (PRa 1.12; 95%CI 1.06-1.18).

In addition, engaging in physical activity for >300 minutes per week (PRa 0.85; 95%CI 0.80-0.89); frequently consuming fruit (PRa 0.86; 95%CI 0.81-0.92); having one or more friends (PRa 0.80; 95% CI 0.74-0.86); and having sought any health service (PRa 0.92; 95%CI 0.88-0.96) remained associated with the outcome after adjustment. Missing classes for health reasons for 1-3 days (PRa 1.31; 95%CI 1.24-1.39) or for 4 or more days (PRa 1.64; 95%CI 1.54-1.74) was also associated with poor SAH. Moreover, reporting feeling worried (PRa 1.09; 95%CI 1.02-1.15), sad (PRa 1.33; 95%CI 1.24-1.41), that nobody cares about them (PRa 1.22; 95%CI 1.16-1.29), irritated, nervous, or moody (PRa 1.79; 95%CI 1.10-1.25), and that life is not worth living (PRa 1.38; 95%CI 1.32-1.45) remained associated with the outcome after adjustment (Table 2).

**Table 2.** Adjusted model of poor self-assessed health among Brazilian school adolescents, according to associated factors, with corresponding 95% confidence intervals. National School Health Survey, Brazil, 2019.

	Poor	
	PRa	p-value
Gender		
Female	1	<0.001
Male	0.75 (0.72-0.79)	
Age (years)		
13 to 15	1	<0.001
16 or 17	1.13 (1.08-1.18)	
Race/color		
White	1	
Black	1.08 (1.01-1.14)	0.014
Brown	1.10 (1.05-1.16)	<0.001
Others	1.13 (1.04-1.22)	0.002
Type of school		
Private		
Public		
Mother’s educational level		
None/Primary (incomplete/complete)		
Secondary (incomplete/complete)		
Higher education (incomplete/complete)		
Regular breakfast consumption (times per week)		
Does not have breakfast	1	<0.001
1 to 4		
5 or more	0.84 (0.80-0.87)	
Ever smoked		
No		
Yes		
Ever drank alcohol		
No	1	<0.001
Yes	1.12 (1.07-1.18)	
Drug experimentation		
No		
Yes		
Body image		
Normal	1	
overweight	1.34 (1.27-1.42)	<0.001
Underweight	1.12 (1.06-1.18)	<0.001
Soft drink consumption (>5 times/week)		
No		
Yes		
Frequent fruit consumption (>5 times/week)		
No	1	<0.001
Yes	0.86 (0.81-0.92)	
Regular physical activity (minutes per week)		
<300	1	<0.001
>300	0.85 (0.80-0.89)	
Friends		
None	1	
1 or more	0.80 (0.74-0.86)	
Sought any health service		
No	1	<0.001
Yes	0.92 (0.88-0.96)	
Missed class due to health problems (days)		
No	1	
1 to 3	1.31 (1.24-1.39)	<0.001
4 or more	1.64 (1.54-1.74)	<0.001
Felt worried in the past 30 days		
No	1	0.004
Yes	1.09 (1.02-1.15)	
Felt sad in the past 30 days		
No	1	<0.001
Yes	1.33 (1.24-1.41)	
Felt that no one cares about you in the past 30 days		
No	1	<0.001
Yes	1.22 (1.16-1.29)	
Felt irritated, nervous, or in a bad mood in the past 30 days		
No	1	<0.001
Yes	1.79 (1.10-1.25)	
Felt that life is not worth living in the past 30 days		
No	1	<0.001
Yes	1.38 (1.32-1.45)	

95%: 95% Confidence Interval; PRa: adjusted prevalence ratio.

## DISCUSSION

This study revealed that approximately 33% of Brazilian adolescents self-assessed their health status as poor. Individual, behavioral, and psychosocial characteristics associated with poor self-assessment included being 16 or 17 years old; identifying as Black, Brown, or another race/ethnicity; consuming alcoholic beverages; perceiving oneself as overweight or underweight; missing school due to health reasons; feeling worried, sad, uncared for, irritated, nervous, or moody; and believing that life is not worth living.

Conversely, lower likelihoods of poor self-assessed health were observed among male adolescents, those who regularly consumed fruit and engaged in physical activity, had friends, sought health services, and maintained the habit of eating breakfast regularly.

The global prevalence of poor SAH among adolescents ranges from 1.2% to 38%, with higher frequencies observed among girls^19^. In the present study, a similar pattern was noted, as males were less likely to report poor SAH, corroborating previous findings in the national literature. Although adolescent girls tend to demonstrate greater concern for their health and seek health services more frequently, thereby becoming more informed about health-disease processes, their SAH remains more negative^4^ ^,^ ^10^. One possible explanation is that girls are more attuned to bodily changes and more able to recognize health risks^14^.

In recent years, the proportion of adolescents perceiving their body image as conforming to normal standards has decreased, with an increase observed among boys who perceive themselves as thin or very thin and girls who perceive themselves as overweight or very overweight^20^. This body dissatisfaction, influenced by factors such as social media, traditional media, and sociodemographic characteristics, can impact lifestyle habits and psychological well-being^20^ ^,^ ^21^. In the present study, inadequate body perception was associated with poor SAH, particularly among adolescents who perceive themselves as outside their ideal weight range, consistent with previous findings^22^. Furthermore, a prevalence of 27.6% of negative SAH was noted among adolescents with distorted body perception^21^. This differentiated pattern between boys and girls reflects cultural and social norms that shape young people’s relationship with their bodies and their health^19^ ^,^ ^20^.

Adolescents tend to prioritize ultra-processed foods, such as fried foods, sweets, and soft drinks, over healthier options like fruits^21^. Unbalanced diets have been associated with poorer self-perceived health^21^. In the present study, regular breakfast consumption was associated with a lower likelihood of poor SAH, highlighting the importance of this habit as an indicator of a healthy lifestyle. Previous research also indicates that regular breakfast consumption contributes to cognitive and academic performance, quality of life, well-being, and the reduction of morbidity risk factors^8^ ^,^ ^23^. Therefore, regular breakfast consumption can be considered a protective factor for perceived health among adolescents.

Furthermore, this study demonstrated that regular physical activity and frequent fruit consumption were associated with a lower likelihood of poor SAH. Notably, healthy lifestyle habits, such as engaging in physical exercise and consuming fruit daily^8^, have been identified as positive factors influencing adolescents’ self-perception of health, as observed in the present study.

There has been a significant increase in mental distress among adolescents in recent years^22^. Similar to this study, other research has identified that frequent feelings of sadness, a sense of worthlessness, and the perception that no one cares are associated with a negative self-perception of health^20^. These feelings were more prevalent among female adolescents, reinforcing the findings of this research^20^. This scenario is concerning, as adolescence is a vulnerable phase for the emergence of depressive and anxious symptoms.

This study identified an association between race/color and poor SAH among adolescents. This finding is consistent with national evidence highlighting the influence of racial inequalities on individuals’ health perceptions^4^ ^,^ ^24^ ^,^ ^25^. Studies conducted in different Brazilian populations have shown that mixed-race individuals tend to report worse self-reported health indicators compared to white individuals^4^ ^,^ ^24^ ^,^ ^25^. These results reflect the impact of structural inequalities and social conditions on the health-disease process, affecting not only objective health outcomes but also the subjective perception of well-being^4^ ^,^ ^24^ ^,^ ^25^.

The literature indicates that SAH tends to become more negative with increasing age^14^, a pattern confirmed in the present study: adolescents aged 16-17 years reported worse self-perceived health compared to those aged 13-15 years. This difference may be related to cognitive maturation, which enables older adolescents to conceptualize health more comprehensively, taking into account not only the absence of disease but also physical, emotional, and social aspects^14^. Such maturation may also influence how adolescents perceive and evaluate their own health over time^10^.

The limitations of this study include potential recall bias and misinterpretation arising from the use of a self-administered questionnaire. The sample was restricted to enrolled students, thereby excluding out-of-school adolescents, who may be more vulnerable. As a cross-sectional study, causal relationships cannot be established. Nonetheless, PeNSE includes schools from diverse regions, including hard-to-reach areas, ensuring national representativeness and allowing the generalization of the findings to Brazilian adolescents.

Therefore, in addition to sociodemographic and behavioral factors, mental health was also found to be associated with the outcome, highlighting the need for further in-depth studies to better understand its determinants and long-term implications.

## Supplementary Materials

Table 1
