# Supplementary material for: Prevalence and factors associated with poor self-assessed health in Brazilian adolescents: National School Health Survey 2019
Source: Rev Bras Epidemiol. 2026 Mar 16;29:e260007. doi: 10.1590/1980-549720260007 (PMC12995339; doi:10.1590/1980-549720260007)
Supplement: Table 1 [file 1980-5497-rbepid-29-e260007-sppl.pdf]

**Tabela 1S. Modelos brutos de Razão de Prevalência da autoavaliação de saúde ruim entre adolescentes escolares brasileiros, segundo fatores associados, com respectivos intervalos de confiança de 95%. Pesquisa Nacional de Saúde do Escolar, Brasil, 2019.**

| | **Razoável/ruim/muito ruim** | |
|---|---|---|
| | **RP Não ajustado** | ***p*** |
| **Sexo** | | |
| Feminino | 1 | |
| Masculino | 0,43 (0,38-0,49) | **<0,001** |
| **Idade** | | |
| 13 a 15 anos | 1 | |
| 16 ou 17 anos | 1,41 (1,24-1,60) | **<0,001** |
| **Raça/cor** | | |
| Branca | 1 | |
| Preta | 1,11 (0,93-1,33) | 0,233 |
| Parda | 1,04 (0,91-1,18) | 0,518 |
| Outros | 1,15 (0,93-1,43) | **0,185** |
| **Tipo de escola** | | |
| Privada | 1 | |
| Pública | 1,11 (1,01-1,23) | **0,030** |
| **Escolaridade da mãe** | | |
| Sem escolaridade/Primário (incompleto/completo) | 1 | |
| Secundário (incompleto/completo) | 0,92 (0,81-1,04) | **0,190** |
| Superior (incompleto/completo) | 1,00 (0,87-1,15) | 0,915 |
| **Toma café da manhã regularmente** | | |
| Não toma | 1 | |
| 1 a 4 vezes na semana | 0,87 (0,69-1,10) | 0,264 |
| 5 ou mais vezes na semana | 0,45 (0,39-0,52) | **<0,001** |
| **Já fumou** | | |
| Não | 1 | |
| Sim | 1,87 (1,65-2,11) | **<0,001** |
| **Já bebeu** | | |
| Não | 1 | |
| Sim | 1,70 (1,48-1,95) | **<0,001** |
| **Experimentação de drogas** | | |
| Não | 1 | |
| Sim | 2,08 (1,79-2,42) | **<0,001** |
| **Imagem corporal** | | |
| Normal | 1 | |
| Gordo | 3,25 (2,84-3,73) | **<0,001** |
| Magro | 1,86 (1,61-2,14) | **<0,001** |
| **Consumo de refrigerantes (>5 vezes na semana)** | | |
| Não | 1 | |

| | | |
|---|---|---|
| Sim | 1,34 (1,12-1,59) | **0,001** |
| **Consumo de frutas frequente (>5 vezes na semana)** | | |
| Não | 1 | |
| Sim | 0,73 (0,63-0,85) | **<0,001** |
| **Atividade física regular** | | |
| <300 minutos semanais | 1 | |
| >300 minutos semanais | 0,69 (0,59-0,82) | **<0,001** |
| **Amigos** | | |
| Não tenho | 1 | |
| 1 ou mais | 0,30 (0,24-0,38) | **<0,001** |
| **Procurou algum serviço de saúde** | | |
| Não | 1 | |
| Sim | 0,92 (0,82-1,04) | 0,208 |
| **Faltar à aula por motivo de saúde** | | |
| Não | 1 | |
| 1 a 3 dias | 1,66 (1,44-1,91) | **<0,001** |
| 4 ou mais dias | 4,69 (3,98-5,54) | **<0,001** |
| **Sentiu-se preocupado(a) nos últimos 30 dias** | | |
| Não | 1 | |
| Sim | 1,10 (0,92-1,31) | 0,265 |
| **Sentiu-se triste nos últimos 30 dias** | | |
| Não | 1 | |
| Sim | 3,22 (2,68-3,86) | **<0,001** |
| **Sentiu que ninguém se preocupa com você nos últimos 30 dias** | | |
| Não | 1 | |
| Sim | 3,19 (2,77-3,67) | **<0,001** |
| **Sentiu-se irritado(a), nervoso(a) ou mal-humorado(a) nos últimos 30 dias** | | |
| Não | 1 | |
| Sim | 2,29 (1,85-2,84) | **<0,001** |
| **Sentiu que a vida não vale a pena ser vivida nos últimos 30 dias** | | |
| Não | 1 | |
| Sim | 4,11 (3,59-4,70) | **<0,001** |

IC95%: intervalo de confiança de 95%; RP: razão de prevalência.
