# Supplementary material for: Prevalence and factors associated with poor self-assessed health in Brazilian adolescents: National School Health Survey 2019
Source: Rev Bras Epidemiol. 2026 Mar 16;29:e260007. doi: 10.1590/1980-549720260007 (PMC12995339; doi:10.1590/1980-549720260007)
Supplement: Translation

# Translation: Prevalência e fatores associados à autoavaliação de saúde ruim em adolescentes brasileiros: Pesquisa Nacional de Saúde do Escolar 2019

**DOI:** 10.1590/1980-549720260007.2

**Authors:** Luís Antônio Batista Tonaco, Bárbara Aguiar Carrato, Leonardo Lemos Pena, Deborah Carvalho Malta

**Affiliations:** I Universidade Federal de Minas Gerais - Belo Horizonte (MG), Brasil.; II Universidade Federal de Minas Gerais, Escola de Enfermagem - Belo Horizonte (MG), Brasil.; III Universidade Federal de Minas Gerais, Escola de Enfermagem, Programa de Pós-Graduação - Belo Horizonte (MG), Brasil.; IV Universidade Federal de Minas Gerais, Escola de Enfermagem, Departamento de Enfermagem Materno-Infantil e Saúde Pública - Belo Horizonte (MG), Brasil.

**Keywords:** Adolescente, Autoavaliação, Nível de saúde, Inquéritos epidemiológicos, Brasil

## Abstract

**Objetivo:**

Estimar a prevalência e os fatores associados à autoavaliação ruim do estado de saúde em escolares brasileiros de 13 a 17 anos.

**Métodos:**

Estudo transversal com dados da Pesquisa Nacional de Saúde do Escolar 2019. Foram calculadas as prevalências e razões de prevalências (brutas e ajustadas) por regressão logística, considerando variáveis associadas à autoavaliação ruim de saúde entre adolescentes brasileiros. As análises foram realizadas no *Stata*, versão 16.

**Resultados:**

As características associadas à autoavaliação de saúde ruim foram: ser do sexo masculino (RPa 0,75; IC95% 0,72-0,79); ter 16 ou 17 anos (RPa 1,13; IC95% 1,08-1,18); de raça/cor preta (RPa 1,08; IC95% 1,01-1,14), parda (RPa 1,10; IC95% 1,05-1,16) e outras (RPa 1,13; IC95% 1,04-1,22); tomar café da manhã (RPa 0,84; IC95% 0,80-0,87); já ter experimentado bebidas alcoólicas (RPa 1,12; IC95% 1,07-1,18); perceber-se gordo (RPa 1,34; IC95% 1,27-1,42) ou magro (RPa 1,12; IC95% 1,06-1,18); praticar atividade física (RPa 0,85; IC95% 0,80-0,89), consumir frutas (RPa 0,86; IC95% 0,81-0,92); ter 1 ou mais amigos (RPa 0,80; IC95% 0,74-0,86); já ter procurado algum serviço de saúde (RPa 0,92; IC95% 0,88-0,96); faltar à aula por motivos de saúde em 1 a 3 dias (RPa 1,31; IC95% 1,24-1,39) ou 4 ou mais dias (RPa 1,64; IC95% 1,54-1,74); sentir-se preocupado(a) (RPa 1,09; IC95% 1,02-1,15), triste (RPa 1,33; IC95% 1,24-1,41), que ninguém se preocupa consigo (RPa 1,22; IC95% 1,16-1,29), irritado(a) (RPa 1,79; IC95% 1,10-1,25) e que a vida não vale a pena ser vivida (RPa 1,38; IC95% 1,32-1,45).

**Conclusão:**

Fatores sociodemográficos, comportamentais e de saúde mental associaram-se ao desfecho.

## INTRODUÇÃO

A Autoavaliação de Saúde (AAS) é amplamente utilizada em estudos e inquéritos populacionais como um importante indicador subjetivo das condições de saúde e bem-estar, por sua simplicidade de aplicação e capacidade de captar percepções individuais sobre o estado de saúde^1^ ^,^ ^2^ ^,^ ^3^ ^,^ ^4^. Embora subjetiva, a AAS constitui um indicador abrangente que reflete tanto a percepção individual do estado de saúde quanto a influência de determinantes sociais, comportamentais e ambientais^1^ ^,^ ^2^ ^,^ ^3^ ^,^ ^4^. Estudos recentes têm demonstrado que a AAS se associa a diversos fatores objetivos e subjetivos, incluindo condições socioeconômicas (escolaridade, renda, ocupação) e aspectos culturais, psicológicos (presença de depressão ou bem-estar subjetivo) e sociais (rede de apoio e relacionamentos interpessoais)^1^ ^,^ ^2^ ^,^ ^3^ ^,^ ^4^.

Uma percepção positiva da própria saúde associa-se a bem-estar psicológico^5^, maior adesão a tratamentos e adoção de comportamentos preventivos, resultando em melhores desfechos em saúde^6^. Em contrapartida, uma autopercepção negativa está relacionada a piores prognósticos, maior consumo de substâncias e adoção de hábitos prejudiciais, ampliando os riscos à saúde^7^ ^,^ ^8^. Assim, a AAS é considerada um importante instrumento para a análise das condições de saúde das populações e subsidia a formulação e implementação de políticas públicas de promoção da saúde^7^.

Nas últimas décadas, a autopercepção de saúde entre adolescentes tem ganhado destaque na investigação dos determinantes do processo saúde-doença^9^ ^,^ ^10^. A adolescência representa um período de intensas transformações físicas, psicológicas e sociais, marcado pela construção da identidade individual e coletiva, o que influencia diretamente a adoção de hábitos e comportamentos que podem se estender à vida adulta^11^ ^,^ ^12^ ^,^ ^13^. Diversos estudos têm demonstrado que adolescentes do sexo feminino, mais velhos, que se percebem fora do peso ideal ou que apresentam sintomas de sofrimento psicológico tendem a relatar pior autoavaliação de saúde; enquanto aqueles que mantêm hábitos saudáveis, como tomar café da manhã regularmente, praticar atividade física e consumir frutas, apresentam melhor percepção da própria saúde^4^ ^,^ ^11^ ^,^ ^12^ ^,^ ^13^ ^,^ ^14^ ^,^ ^15^.

Diversos fatores têm sido associados a uma percepção negativa da própria saúde nessa faixa etária, incluindo comportamentos de risco como sedentarismo, tabagismo, consumo de álcool e outras substâncias, além da exposição à violência escolar^11^ ^,^ ^13^ ^,^ ^14^. Aspectos sociodemográficos, como sexo, idade, convivência com os pais, realização de refeições em família e frequência do café da manhã, também apresentam associação com maiores prevalências de autopercepção negativa de saúde entre adolescentes^4^ ^,^ ^15^.

Em contextos internacionais, resultados semelhantes têm sido observados em diferentes países, indicando consistência nas associações entre autopercepção negativa de saúde e fatores como estresse, sintomas depressivos, baixo nível de atividade física e hábitos alimentares inadequados^16^. Na Coreia do Sul, por exemplo, um estudo com estudantes do ensino fundamental e médio evidenciou que a autopercepção negativa da saúde esteve significativamente associada à presença de estresse, ideação e tentativas suicidas, além de baixa atividade física e omissão de refeições como o café da manhã^16^. Esses achados são coerentes com o observado em outras populações, reforçando que, independentemente do contexto sociocultural, determinantes psicossociais e comportamentais desempenham papel central na forma como adolescentes percebem sua saúde.

No contexto brasileiro, a Pesquisa Nacional de Saúde do Escolar (PeNSE) tem incluído a AAS em seus questionários, reconhecendo sua relevância para compreender a percepção de saúde entre adolescentes^4^. Estudo com dados da PeNSE 2015 identificou que meninas, adolescentes de 16 e 17 anos, de raça/cor preta ou parda e aqueles com comportamentos de risco apresentaram maior chance de AAS ruim, enquanto a realização regular do café da manhã e a prática de atividade física estiveram associadas a menor chance de autopercepção negativa, embora a definição da variável desfecho tenha diferido daquela utilizada no presente estudo^4^. Outras pesquisas nacionais também evidenciaram associações entre a AAS e diferentes características sociodemográficas e de estilo de vida, como qualidade de vida^4^, sexo^4^ ^,^ ^10^, idade^4^ ^,^ ^10^, raça/cor^4^, hábitos de vida^4^ ^,^ ^10^, procura por serviços de saúde^4^ e escolaridade materna^4^ ^,^ ^15^.

Apesar dos avanços, ainda existe uma lacuna na literatura científica quanto à avaliação da autopercepção negativa de saúde entre adolescentes brasileiros em estudos de abrangência nacional e com dados atualizados. A mensuração da AAS em inquéritos populacionais, por meio de escalas padronizadas, permite captar de forma representativa a percepção de saúde da população, fornecendo subsídios para a compreensão dos determinantes sociais e comportamentais que influenciam o bem-estar^4^.

Dessa forma, considerando a importância da AAS como indicador das condições de saúde e do bem-estar psicológico de adolescentes, o presente estudo teve como objetivo estimar a prevalência e os fatores associados à autoavaliação ruim do estado de saúde em escolares brasileiros de 13 a 17 anos, utilizando dados da Pesquisa Nacional de Saúde do Escolar 2019.

## MÉTODOS

Trata-se de um estudo transversal com dados da PeNSE, edição 2019. Os dados são de domínio público, disponíveis no site do Instituto Brasileiro de Geografia e Estatística (IBGE)^17^.

A PeNSE é um inquérito nacional conduzido pelo IBGE, em parceria com o Ministério da Saúde e com o apoio do Ministério da Educação^17^ ^,^ ^18^, e investiga fatores de risco e proteção à saúde de adolescentes brasileiros, coletando dados sobre hábitos como atividade física, alimentação, uso de substâncias, saúde bucal, comportamento sexual e violência^17^ ^,^ ^18^. A primeira edição da PeNSE foi realizada em 2009, sendo posteriormente repetida nos anos de 2012, 2015 e 2019^17^ ^,^ ^18^.

A amostra final foi composta pelos estudantes pertencentes às turmas selecionadas. A pesquisa utilizou como base o Cadastro de Escolas Públicas e Privadas do Instituto Nacional de Estudos e Pesquisas Educacionais Anísio Teixeira (Inep), garantindo a representatividade dos seguintes níveis geográficos: Brasil, grandes regiões, Unidades da Federação (UF), capitais e o Distrito Federal^17^ ^,^ ^18^.

Foram selecionados para responder ao questionário todos os alunos regularmente matriculados e frequentes nas aulas do 7^o^ ao 9^o^ ano do ensino fundamental e da 1^a^ à 3^a^ série do ensino médio, em escolas públicas e privadas, nos turnos matutino, vespertino e noturno^17^ ^,^ ^18^. Também foram incluídos os alunos matriculados em cursos técnicos integrados ao ensino médio e cursos de formação para o magistério^17^ ^,^ ^18^. Apenas os estudantes presentes no dia da coleta participaram do estudo. Escolas com menos de 20 alunos matriculados foram excluídas^17^ ^,^ ^18^.

A amostra foi obtida por meio de amostragem probabilística em múltiplos estágios (conglomerados), com estratificação por UF, localização (urbana ou rural) e tipo de escola (pública ou privada) ^17^ ^,^ ^18^.

O processo de seleção ocorreu em 3 estágios:

1. Seleção probabilística das escolas em cada estrato;
2. Seleção aleatória de turmas nas escolas selecionadas; e
3. Inclusão de todos os estudantes presentes nas turmas selecionadas no momento da coleta^17^ ^,^ ^18^.

Mais detalhes metodológicos sobre a pesquisa e o plano amostral podem ser encontrados em publicações anteriores^17^ ^,^ ^18^.

Para esta análise, o desfecho investigado foi a autoavaliação do estado de saúde, com base na pergunta presente no questionário da PeNSE 2019: “*Como você classificaria seu estado de saúde?*”. As opções de resposta incluíram: muito bom, bom, regular, ruim e muito ruim. Para a construção da variável desfecho (autoavaliação ruim de saúde), as respostas foram agregadas em duas categorias: a primeira agregou as opções “muito bom”, “bom” e “regular”, enquanto a segunda agrupou “ruim” e “muito ruim”.

A mensuração da AAS é comumente realizada por meio de um único item, com escala ordinal de cinco pontos, semelhante à utilizada na PeNSE. Termos como *self-rated health*, *self-assessed health* e *self-reported health* são frequentemente empregados na literatura de forma equivalente para se referir a essa medida subjetiva de percepção global de saúde^4^ ^,^ ^17^ ^,^ ^18^. A questão utilizada para mensurar a AAS é amplamente empregada em inquéritos populacionais e apresenta evidências consolidadas de validade e confiabilidade em diferentes contextos^4^ ^,^ ^17^ ^,^ ^18^.

As variáveis independentes, por sua vez, foram selecionadas com base na literatura, a qual indica que a autopercepção de saúde está associada a fatores sociodemográficos, grau de escolaridade, raça/cor, características familiares, hábitos de vida, comportamentos, procura por serviços de saúde e fatores relacionados à saúde mental^4^. Dessa forma, as variáveis independentes foram distribuídas conforme descrito abaixo.

As características sociodemográficas incluíram: sexo (feminino e masculino), idade em anos (13 a 15, e 16 ou 17 anos), raça/cor (branca, preta, parda e outros), tipo de escola (pública e privada), e escolaridade da mãe (sem escolaridade, primário [incompleto/completo], secundário [incompleto/completo] e superior [incompleto/completo]). As características familiares consideraram se o indivíduo morava com a mãe (sim e não), morava com o pai (sim e não), fazia refeição com o responsável (não, 2 vezes ou menos na semana, 3 a 4 vezes semanais, e 5 ou mais vezes na semana).

Quanto às características sobre hábitos de vida e comportamentos, considerou-se se o adolescente tomava café da manhã regularmente (não tomava, 5 ou mais vezes na semana, 1 a 4 vezes na semana), já experimentou cigarro (não e sim), já experimentou bebidas alcoólicas (não e sim), já experimentou drogas na vida (não e sim), consumia refrigerantes >5 vezes na semana (não e sim), consumia frutas >5 vezes na semana (não e sim), realizava atividade física regular (<300 minutos semanais e >300 minutos semanais) e se tinha relação sexual (não e sim).

Além disso, considerou-se se o adolescente já faltou à aula por algum motivo de saúde (não, 1 a 3 dias e 4 ou mais dias), como vê sua imagem corporal (normal, gordo e magro) e se já procurou algum serviço de saúde (não e sim). Quanto às características sobre saúde mental, por fim, considerou-se se o adolescente tem amigos (não tenho, e 1 ou mais); sentiu-se preocupado nos últimos 30 dias (não e sim); sentiu-se triste nos últimos 30 dias (não e sim); sentiu que ninguém se preocupa com ele(a) nos últimos 30 dias (não e sim); sentiu-se irritado(a), nervoso(a) ou mal-humorado(a) nos últimos 30 dias (não e sim); e se sentiu que a vida não vale a pena ser vivida nos últimos 30 dias (não e sim).

Inicialmente, foram realizadas análises descritivas para estimar as prevalências e os seus respectivos intervalos de confiança de 95% (IC95%), segundo as variáveis independentes mencionadas.

Em seguida, para investigar os fatores associados ao desfecho analisado (autoavaliação ruim de saúde), foi realizada inicialmente uma análise bivariada, estimando-se os valores não ajustados de razão de prevalência (RP) e seus respectivos IC95% por meio de regressão logística simples. Em seguida, foi aplicado um modelo de regressão logística multivariada, incluindo as variáveis de interesse que apresentaram p<0,20 na análise bivariada, conforme critérios estatísticos. No modelo multivariado final (RPa), foram mantidas apenas as variáveis estatisticamente significativas (p<0,05). Para avaliar o ajuste do modelo final, foi aplicado o teste de Hosmer-Lemeshow.

Todas as análises foram conduzidas com o uso do módulo svy do software *Stata Statistical Software*, versão 16, que permite a incorporação adequada dos pesos amostrais, estratos e conglomerados.

O presente estudo utilizou dados secundários, dispensando, portanto, a necessidade de submissão ao Comitê de Ética em Pesquisa. Contudo, a edição de 2019 da PeNSE obteve aprovação da Comissão Nacional de Ética em Pesquisa com Seres Humanos (Conep), sob o parecer n^o^ 3.249.268^17^ ^,^ ^18^.

## DECLARAÇÃO DE DISPONIBILIDADE DE DADOS

Todo o conjunto de dados que dá suporte aos resultados deste estudo está disponível mediante solicitação ao autor correspondente.

## RESULTADOS

Do total de 103.177 adolescentes incluídos na base de dados, 100.221 (97,1%) apresentaram informações completas sobre as variáveis de interesse e foram incluídos na análise final. Foram excluídos 2.956 casos (2,9%) devido à ausência de dados em uma ou mais variáveis.

A amostra deste estudo foi composta majoritariamente por estudantes do sexo feminino (52,17%), de 13 a 15 anos (64%) e pardos (43,01%). A maioria estava matriculada em escolas públicas (51,73%) e tinha mães com escolaridade superior incompleto/completo (46,91%). Quanto aos hábitos, 58,95% dos estudantes relataram tomar café da manhã regularmente, enquanto 79,63% nunca experimentaram cigarro, 63,94% já experimentaram bebidas alcoólicas, e 87,59% nunca haviam experimentado drogas. O consumo frequente de refrigerantes foi relatado por 15,92%, e de frutas por 28,42%. Quanto à atividade física, 71,58% praticavam menos de 300 minutos semanais (Tabela 1).

**Tabela 1.** Distribuição e prevalência de autoavaliação de saúde ruim entre adolescentes escolares brasileiros, segundo fatores associados, com respectivos Intervalos de Confiança de 95%. Pesquisa Nacional de Saúde do Escolar, Brasil, 2019.

	Distribuição (%)	Regular/ruim/muito ruim	Valor p
		n (%)	
Total		32,80 (32,51-33,08)	
Sexo			
Feminino	52,17	39,55 (38,62-40,50)	<0,0001
Masculino	47,82	20,96 (20,17-21,77)	
Idade (anos)			
13 a 15	64,00	28,15 (27,27-29,04)	<0,0001
16 ou 17	35,99	35,69 (34,75-36,63)	
Raça/cor			
Branca	40,32	28,93 (27,81-30,07)	0,0008
Preta	10,47	30,43 (28,79-32,13)	
Parda	43,01	31,86 (30,75-33)	
Outras	6,18	32,99 (30,42-35,68)	
Tipo de escola			
Pública	51,73	31,10 (30,33-31,88)	<0,0001
Privada	48,26	28,11 (27,33-28,91)	
Escolaridade da mãe			
Sem escolaridade/Primário (incompleto/completo)	22,5	30,88 (29,80-31,98)	0,0019
Secundário (incompleto/completo)	30,57	31,97 (30,80-33,17)	
Superior (incompleto/completo)	46,91	28,84 (27,62-30,10)	
Toma café da manhã regularmente			
Não toma	32,42	39,60 (38,43-40,78)	<0,0001
1 a 4 vezes na semana	8,79	34,61 (32,23-37,07)	
5 ou mais vezes na semana	58,95	25,16 (24,43-25,91)	
Já fumou			
Não	79,63	28,07 (27,31-28,84)	<0,0001
Sim	20,36	39,70 (38,23-41,20)	
Já bebeu			
Não	36,05	22,75 (21,89-23,64)	<0,0001
Sim	63,94	35,22 (34,30-36,14)	
Experimentação de drogas			
Não	87,59	29,11 (28,53-29,80)	<0,0001
Sim	12,4	41,31 (39,37-43,28)	
Imagem corporal			
Normal	47,91	24,35 (23,41-25,32)	<0,0001
Gordo	23,30	44,28 (42,57-46)	
Magro	28,77	31,50 (30,31-32,72)	
Consumo de refrigerantes (>5 vezes na semana)			
Não	84,07	30,27 (29,48-31,07)	0,019
Sim	15,92	32,56 (30,95-34,22)	
Consumo de frutas frequente (>5 vezes na semana)			
Não	71,57	32,87 (31,98-33,77)	<0,0001
Sim	28,42	25,05 (23,77-26,37)	
Atividade física regular (minutos semanais)			
<300	71,58	33,54 (32,69-34,40)	<0,0001
>300	28,41	23,71 (22,70-24,75)	
Amigos			
Não tenho	3,20	44,69 (41,23-48,21)	<0,0001
1 ou mais	96,79	30,10 (29,40-30,80)	
Procurou algum serviço de saúde			
Não	35,95	29,19 (28,13-30,28)	0,0002
Sim	64,04	31,73 (30,91-32,55)	
Faltou à aula por motivo de saúde			
Não	37,72	23,40 (22,54-24,29)	<0,0001
1 a 3 dias	52,31	33,89 (32,79-35,01)	
4 ou mais dias	9,95	47,84 (45,39-50,30)	
Sentiu-se preocupado(a) nos últimos 30 dias			
Não	15,53	23,63 (22,25-25,07)	<0,0001
Sim	84,46	32,53 (31,81-33,26)	
Sentiu-se triste nos últimos 30 dias			
Não	31,87	16,19 (15,35-17,06)	<0,0001
Sim	68,12	38,12 (37,30-38,94)	
Sentiu que ninguém se preocupa com você nos últimos 30 dias			
Não	45,88	18,10 (17,05-19,21)	<0,0001
Sim	54,11	35,07 (34,33-35,82)	
Sentiu-se irritado(a), nervoso(a) ou mal-humorado(a) nos últimos 30 dias			
Não	23,09	18,10 (17,05-19,21)	<0,0001
Sim	76,9	35,07 (34,33-35,82)	
Sentiu que a vida não vale a pena ser vivida nos últimos 30 dias			
Não	63,89	21,43 (20,70-22,18)	<0,0001
Sim	36,1	45,82 (44,78-46,85)	

IC95%: intervalo de confiança de 95%.

A imagem corporal predominante foi normal (47,91%). A quase totalidade relatou ter um ou mais amigos (96,79%), 84,46% já procuraram algum serviço de saúde, e 52,31% tinham faltado à escola 4 ou mais dias por motivo de saúde. Em relação ao bem-estar psicológico, 84,46% dos adolescentes relataram sentir-se preocupados(as); 68,12% sentiram-se tristes; 54,11% sentiram que ninguém se preocupava com eles; 76,90% sentiram-se irritados(as), nervosos(as) ou mal-humorados(as); e 36,10% sentiram que a vida não valia a pena ser vivida (Tabela 1).

Dos escolares brasileiros, 32,80% (IC95% 32,51-33,08) relataram autoavaliação ruim do estado de saúde, prevalência que se mostrou mais elevada entre o sexo feminino (39,55%; IC95% 38,62-40,50), entre os escolares de 16 ou 17 anos (35,69%; IC95% 34,75-36,63), de outra raça/cor (32,99%; IC95% 30,42-35,68), matriculados em escolas públicas (31,10%; IC95% 30,33-31,88) e cujas mães tinham escolaridade secundária incompleta/completa (31,97%; IC95% 30,87-33,17). Também foram observadas maiores prevalências entre os escolares que não tomavam café da manhã regularmente (39,60%; IC95% 38,43-40,78), bem como entre os que já experimentaram cigarro (39,70%; IC95% 38,23-41,20) e aqueles que já consumiram bebidas alcoólicas (35,22%; IC95% 34,30-36,14) (Tabela 1).

Observou-se, ainda, maiores prevalências entre os escolares que já experimentaram drogas (41,31%; IC95% 39,37-43,28), que se consideravam gordos (44,28%; IC95% 42,57-46,00), que consumiam refrigerantes regularmente (32,56%; IC95% 30,95-34,22) e não consumiam frutas com a mesma frequência (32,87%; IC95% 31,98-33,77), que faziam atividade física <300 minutos semanais (33,54%; IC95% 32,69-34,40), bem como entre aqueles que referiram não ter amigos (44,69%; IC95% 41,23-48,21). Além disso, maiores prevalências foram encontradas entre os escolares que já procuraram algum serviço de saúde (31,73% IC95% 30,91-32,55) e que já faltaram à aula por motivos de saúde em 4 ou mais dias na semana (47,84%; IC95% 45,39-50,30) (Tabela 1).

Ademais, observou-se maiores prevalências entre os escolares que, nos últimos 30 dias, relataram sentir-se preocupados(as) (32,53%; IC95% 31,81-33,26); tristes (38,12%; IC95% 37,30-38,94); que ninguém se preocupa com eles (35,07%; IC95% 34,33-35,82); irritados(as), nervosos(as) ou mal-humorados(as) (35,07%; IC95% 34,33-35,82); e que a vida não vale a pena ser vivida (45,82%; IC95% 44,78-46,85) (Tabela 1).

Na Tabela 1 do Material Suplementar constam as estimativas do modelo bruto (Tabela 1S). As características dos escolares que se mantiveram associadas à autoavaliação de saúde ruim após o ajuste por todas as variáveis do modelo foram: ser do sexo masculino (RPa 0,75; IC95% 0,72-0,79); ter 16 ou 17 anos (RPa 1,13; IC95% 1,08-1,18), raça/cor preta (RPa 1,08; IC95% 1,01-1,14), parda (RPa 1,10; IC95% 1,05-1,16) e outras (RPa 1,13; IC95% 1,04-1,22); tomar café da manhã 5 ou mais vezes na semana (RPa 0,84; IC95% 0,80-0,87); já ter experimentado bebidas alcoólicas (RPa 1,12; IC95% 1,07-1,18); e perceber-se gordo (RPa 1,34; IC95% 1,27-1,42) ou magro (RPa 1,12; IC95% 1,06-1,18).

Somado a isso, praticar atividade física >300 minutos semanais (RPa 0,85; IC95% 0,80-0,89); consumir frutas frequentemente (RPa 0,86; IC95% 0,81-0,92); ter 1 ou mais amigos (RPa 0,80; IC95% 0,74-0,86); já ter procurado algum serviço de saúde (RPa 0,92; IC95% 0,88-0,96); faltar à aula por motivos de saúde em 1 a 3 dias (RPa 1,31; IC95% 1,24-1,39) ou 4 ou mais dias (RPa 1,64; IC95% 1,54-1,74); sentir-se preocupado(a) (RPa 1,09; IC95% 1,02-1,15), triste (RPa 1,33; IC95% 1,24-1,41), que ninguém se preocupa consigo (RPa 1,22; IC95% 1,16-1,29), irritado(a), nervoso(a) ou mal-humorado(a) (RPa 1,79; IC95% 1,10-1,25) e que a vida não vale a pena ser vivida (RPa 1,38; IC95% 1,32-1,45) estiveram associadas ao desfecho após ajuste (Tabela 2).

**Tabela 2.** Modelo ajustado de autoavaliação de saúde ruim entre adolescentes escolares brasileiros, segundo fatores associados, com respectivos intervalos de confiança de 95%. Pesquisa Nacional de Saúde do Escolar, Brasil, 2019.

	Ruim	
	RPa	Valor p
Sexo		
Feminino	1	<0,001
Masculino	0,75 (0,72-0,79)	
Idade (anos)		
13 a 15	1	<0,001
16 ou 17	1,13 (1,08-1,18)	
Raça/cor		
Branca	1	
Preta	1,08 (1,01-1,14)	0,014
Parda	1,10 (1,05-1,16)	<0,001
Outras	1,13 (1,04-1,22)	0,002
Tipo de escola		
Privada		
Pública		
Escolaridade da mãe		
Sem escolaridade/primário (incompleto/completo)		
Secundário (incompleto/completo)		
Superior (incompleto/completo)		
Toma café da manhã regularmente (vezes na semana)		
Não toma	1	<0,001
1 a 4		
5 ou mais	0,84 (0,80-0,87)	
Já fumou		
Não		
Sim		
Já bebeu		
Não	1	<0,001
Sim	1,12 (1,07-1,18)	
Experimentação de drogas		
Não		
Sim		
Imagem corporal		
Normal	1	
Gordo	1,34 (1,27-1,42)	<0,001
Magro	1,12 (1,06-1,18)	<0,001
Consumo de refrigerantes (>5 vezes na semana)		
Não		
Sim		
Consumo de frutas frequente (>5 vezes na semana)		
Não	1	<0,001
Sim	0,86 (0,81-0,92)	
Atividade física regular (minutos semanais)		
<300	1	<0,001
>300	0,85 (0,80-0,89)	
Amigos		
Não tenho	1	
1 ou mais	0,80 (0,74-0,86)	
Procurou algum serviço de saúde		
Não	1	<0,001
Sim	0,92 (0,88-0,96)	
Faltou à aula por motivo de saúde (dias)		
Não	1	
1 a 3	1,31 (1,24-1,39)	<0,001
4 ou mais	1,64 (1,54-1,74)	<0,001
Sentiu-se preocupado(a) nos últimos 30 dias		
Não	1	0,004
Sim	1,09 (1,02-1,15)	
Sentiu-se triste nos últimos 30 dias		
Não	1	<0,001
Sim	1,33 (1,24-1,41)	
Sentiu que ninguém se preocupa com você nos últimos 30 dias		
Não	1	<0,001
Sim	1,22 (1,16-1,29)	
Sentiu-se irritado(a), nervoso(a) ou mal-humorado(a) nos últimos 30 dias		
Não	1	<0,001
Sim	1,79 (1,10-1,25)	
Sentiu que a vida não vale a pena ser vivida nos últimos 30 dias		
Não	1	<0,001
Sim	1,38 (1,32-1,45)	

IC95%: Intervalo de Confiança de 95%; RPa: Razão de Prevalência Ajustada.

## DISCUSSÃO

Este estudo evidenciou que aproximadamente 33% dos adolescentes brasileiros autoavaliaram seu estado de saúde como ruim. Foram associadas à autoavaliação ruim características individuais, comportamentais e psicossociais, como ter 16 ou 17 anos; ser de raça/cor preta, parda ou outras; consumo de bebidas alcoólicas; perceber-se gordo ou magro; faltar à aula por motivo de saúde; sentir-se preocupado(a), triste, que ninguém se preocupa consigo, irritado(a), nervoso(a) e mal-humorado(a); e considerar que a vida não vale a pena ser vivida.

Por outro lado, verificou-se menores chances de autoavaliação ruim entre adolescentes do sexo masculino, consumo de frutas e atividade física regular, ter amigos, procura por serviços de saúde e entre aqueles que mantêm o hábito de tomar café da manhã regularmente.

A prevalência global de AAS ruim entre adolescentes pode variar de 1,2% a 38%, com maior frequência entre meninas^19^. No presente estudo essa diferença também foi observada, uma vez que o sexo masculino apresentou menor chance de relatar AAS ruim, confirmando achados prévios na literatura nacional. Embora as adolescentes demonstrem maior preocupação com a saúde e procurem mais os serviços de saúde, o que as torna mais informadas sobre os processos saúde-doença, sua AAS permanece mais negativa^4^ ^,^ ^10^. Uma possível explicação é que meninas percebem mais as mudanças no corpo e reconhecem melhor riscos à saúde^14^.

Nos últimos anos tem-se observado uma diminuição na proporção de adolescentes que percebem sua imagem corporal nos padrões considerados normais, com aumento de meninos que se veem como magros ou muito magros e meninas que se percebem como gordas ou muito gordas^20^. Essa insatisfação corporal, influenciada por fatores como redes sociais, mídia e aspectos sociodemográficos, pode afetar hábitos de vida e o bem-estar psicológico^20^ ^,^ ^21^. Neste estudo, a percepção corporal inadequada esteve associada à AAS ruim, especialmente entre adolescentes que se veem fora do peso ideal, em linha com achados anteriores^22^. Destaca-se, ainda, uma prevalência de 27,6% de ASS negativa entre adolescentes com percepção corporal distorcida^21^. Essa manifestação diferenciada entre meninos e meninas reflete padrões culturais e sociais que moldam a relação dos jovens com o próprio corpo e a sua saúde^19^ ^,^ ^20^.

Os adolescentes tendem a priorizar alimentos ultraprocessados, como frituras, doces e refrigerantes, em detrimento de opções mais saudáveis, como frutas^21^. A alimentação desbalanceada tem sido associada a uma pior autopercepção da saúde^21^. Neste estudo identificou-se que o hábito de tomar café da manhã regularmente esteve associado a uma menor chance de AAS ruim, o que reforça a importância dessa prática como indicador de estilo de vida saudável. Pesquisas anteriores também apontam que o consumo regular do desjejum contribui para o desempenho cognitivo e acadêmico, na qualidade de vida, no bem-estar e nos fatores de risco de morbidade^8^ ^,^ ^23^. Assim, a regularidade no café da manhã pode ser considerada um fator protetor para a saúde percebida entre adolescentes.

Além disso, este estudo evidenciou que a prática regular de atividade física e a ingestão frequente de frutas estiveram associadas a menor chance de uma AAS ruim. Destaca-se que hábitos de vida saudáveis, tais como a prática de exercícios físicos e o consumo diário de frutas^8^, têm sido identificados como um fator positivo para a percepção da própria saúde, conforme aqui observado.

Observa-se um aumento significativo do sofrimento mental entre adolescentes nos últimos anos^22^. Assim como neste estudo, outras pesquisas identificaram que sentimentos frequentes de tristeza, sensação de desvalorização da vida e a percepção de que ninguém se importa estão associados a uma autopercepção negativa da saúde^20^. Esses sentimentos foram mais prevalentes entre adolescentes do sexo feminino, o que reforça os achados desta pesquisa^20^. Esse cenário é preocupante, pois a adolescência é uma fase vulnerável ao surgimento de sintomas depressivos e ansiosos.

Neste estudo identificou-se que raça/cor está associada a AAS ruim em adolescentes. Esse achado dialoga com evidências nacionais que apontam a influência das desigualdades raciais na forma como os indivíduos percebem sua própria saúde^4^ ^,^ ^24^ ^,^ ^25^. Estudos realizados em diferentes populações brasileiras demonstraram que pessoas pardas tendem a relatar piores indicadores de saúde autorreferida quando comparadas às brancas^4^ ^,^ ^24^ ^,^ ^25^. Esses resultados refletem o impacto das desigualdades estruturais e das condições sociais que atravessam o processo saúde-doença, repercutindo não apenas em agravos objetivos, mas também na percepção subjetiva do bem-estar^4^ ^,^ ^24^ ^,^ ^25^.

A literatura aponta que a autopercepção da saúde tende a ser mais negativa com o avanço da idade^14^, o que foi confirmado neste estudo: adolescentes entre 16 e 17 anos apresentaram pior autopercepção da saúde em comparação àqueles com idades entre 13 e 15 anos. Essa diferença pode estar relacionada à maturação cognitiva, que leva os adolescentes mais velhos a conceber a saúde de maneira mais abrangente, considerando não apenas a ausência de doenças, mas também aspectos físicos, emocionais e sociais^14^. Esse amadurecimento pode também influenciar a forma como percebem e avaliam sua própria saúde ao longo dos anos^10^.

As limitações deste estudo incluem possível viés de memória e interpretações equivocadas devido ao uso de questionário autoaplicado. A amostra contempla apenas estudantes matriculados, excluindo adolescentes fora da escola, possivelmente mais vulneráveis. Por ser um estudo transversal, não é possível estabelecer causalidade. Ainda assim, a PeNSE abrange escolas de diferentes regiões, incluindo áreas de difícil acesso, garantindo representatividade nacional e permitindo a generalização dos resultados para os adolescentes brasileiros.

Portanto, além dos fatores sociodemográficos e comportamentais, a saúde mental também se mostrou associada ao desfecho estudado, indicando a necessidade de estudos mais aprofundados para compreender melhor seus determinantes e suas implicações ao longo do tempo.

## Material suplementar

Tabela 1
